# The Brain Tracks Multiple Predictions About the Auditory Scene

**DOI:** 10.3389/fnhum.2021.747769

**Published:** 2021-11-03

**Authors:** Kelin M. Brace, Elyse S. Sussman

**Affiliations:** Department of Neuroscience, Albert Einstein College of Medicine, Bronx, NY, United States

**Keywords:** auditory attention, task switching, pattern detection, mismatch negativity (MMN), event-related potentials (ERPs), neural entrainment

## Abstract

The predictable rhythmic structure is important to most ecologically relevant sounds for humans, such as is found in the rhythm of speech or music. This study addressed the question of how rhythmic predictions are maintained in the auditory system when there are multiple perceptual interpretations occurring simultaneously and emanating from the same sound source. We recorded the electroencephalogram (EEG) while presenting participants with a tone sequence that had two different tone feature patterns, one based on the sequential rhythmic variation in tone duration and the other on sequential rhythmic variation in tone intensity. Participants were presented with the same sound sequences and were instructed to listen for the intensity pattern (ignore fluctuations in duration) and press a response key to detected pattern deviants (attend intensity pattern task); to listen to the duration pattern (ignore fluctuations in intensity) and make a button press to duration pattern deviants (attend duration pattern task), and to watch a movie and ignore the sounds presented to their ears (attend visual task). Both intensity and duration patterns occurred predictably 85% of the time, thus the key question involved evaluating how the brain treated the irrelevant feature patterns (standards and deviants) while performing an auditory or visual task. We expected that task-based feature patterns would have a more robust brain response to attended standards and deviants than the unattended feature patterns. Instead, we found that the neural entrainment to the rhythm of the standard attended patterns had similar power to the standard of the unattended feature patterns. In addition, the infrequent pattern deviants elicited the event-related brain potential called the mismatch negativity component (MMN). The MMN elicited by task-based feature pattern deviants had a similar amplitude to MMNs elicited by unattended pattern deviants that were unattended because they were not the target pattern or because the participant ignored the sounds and watched a movie. Thus, these results demonstrate that the brain tracks multiple predictions about the complexities in sound streams and can automatically track and detect deviations with respect to these predictions. This capability would be useful for switching attention rapidly among multiple objects in a busy auditory scene.

## Introduction

It has long been appreciated that the excitability of the cortex oscillates in a rhythmic fashion (Bishop, 1932). Attention is adaptive, capable of following the fluctuations in the rhythmic structure of speech and music in a dynamic fashion (Large and Jones, 1999). Although many different oscillatory bands have been implicated in neuronal processing, this study focused on lower frequency oscillations, particularly related to attention. These low frequency oscillations are inherently present in the brain. The alignment of low frequency oscillations to external stimuli has been posited as a possible method of attentional selection (Lakatos et al., 2008; Schroeder and Lakatos, 2009).

The brain’s response to sound stimulation can reflect the rhythmic structure and is thought to be a mechanism of selective attention (Lakatos et al., 2008; Schroeder and Lakatos, 2009; Calderone et al., 2014). Moreover, entrainment to stimulus presentation rate is positively correlated with behavioral detection (Large and Jones, 1999; Elhilali et al., 2009; Xiang et al., 2010), and expectation of rhythm has been shown to improve behavioral performance (Dowling et al., 1987). There is evidence of primate primary auditory cortical entraining to rhythmic stimuli after the stimuli have ended, indicating that these are not just evoked responses (Lakatos et al., 2013). Amplitude modulation of sounds is also reflected in the cortical response (Draganova et al., 2002). Attention to a target rhythm within a masking sequence can enhance neural entrainment to the target, originating from the auditory cortex. Performance on a target task is correlated with the strength of neural entrainment (Elhilali et al., 2009). Selective entrainment occurs more strongly to the attended rhythm when multiple possible rhythms are present (Costa-Faidella et al., 2017).

In the current study, we recorded an electroencephalogram (EEG) to investigate how the brain entrains to the rhythm of sounds when there are multiple possible rhythmic interpretations that can be extracted from a single sound stream. Specifically, we tested how attention drives entrainment to the two different rhythms by using a switching paradigm that requires a different task goal associated with each distinct rhythm perception.

To further assess processing associated with rhythmic perception, we used two dependent measures of the event-related brain potentials (ERPs), the mismatch negativity (MMN), and the P3b components that reflect processing of the deviant. The MMN, which is generated within auditory cortices (Tiitinen et al., 1993), provides an ideal tool for simultaneously assessing the brain’s response to the attended and the unattended sound rhythms in the sequence (Sussman et al., 2014). Thus, MMN elicitation can be used to assess the representation of different rhythmic regularities maintained in auditory memory (Moldwin et al., 2017). The MMN system represents pattern regularities in a sequence of sounds (the “standard”; Sussman, 2007) and indexes detection of the violation of those regularities (the “deviant”; Schröger et al., 1992; Sussman et al., 1998, 2014; Picton et al., 2000; Näätänen et al., 2001; Takegata et al., 2001; Winkler et al., 2003; Sussman, 2007; Paavilainen, 2013; Pannese et al., 2015). The P3b component was used to evaluate task-related performance. The P3b component is associated with volitional control and its amplitude and latency are affected by task difficulty in dual task situations (Norman and Bobrow, 1975; Isreal et al., 1980; Kok, 2001). For example, task interference is associated with the elicitation of smaller P3b amplitude and longer P3b latency. Thus, the P3b component can be used together with behavioral indices of performance to assess cognitive demands.

The overarching goal of the current study was to gain a better understanding of complex sound perception by investigating the way in which sounds are represented in auditory memory during task performance when multiple rhythmic interpretations can be perceived from one sound stream. The current experiment incorporated elements of a task-switching paradigm along with manipulation of rhythmic attention. The paradigm was inspired by the methodology used in Costa-Faidella et al. (2017). The sound sequences contained two non-overlapping rhythms created by patterns in different tone features, a tone intensity pattern and a tone duration pattern. The task focused on detecting a pattern deviant in the respective feature (intensity pattern deviant or duration pattern deviant). The targets were unique deviants within the intensity and duration patterns to elicit MMN based on its respective standard pattern. In this way, we were able to assess both the brain representation of the standard rhythms by examining neural entrainment to the rhythm of the standard and deviant detection by examining elicitation of the MMN. We predicted that when participants performed repeated trials of the same task, there would be evidence of strong neural entrainment to the target rhythm, and an MMN elicited by the attended pattern deviants. We further predicted that the entrainment to the unattended, irrelevant rhythm (intensity pattern when attending duration, and duration pattern when attending intensity) would be attenuated or absent, which would likely preclude the MMN response. Additionally, because it was a switching paradigm, we expected some behavioral switch cost (e.g., lower hit rate or longer response time) when participants alternated between tasks. Finally, because we set up a competition between stimulus-driven rhythmic perceptions, we expected that there may be neural evidence of competition between the tasks.

## Materials and Methods

### Participants

Twenty-four adults aged 22–41 years (median age 27.5, 11 males) were included in this study. All participants passed a hearing screening (25 dB HL or better for 500, 1,000, 2,000, and 4,000 Hz) and had no self-reported history of psychiatric or neurologic disorder. The study was carried out in accordance with the Code of Ethics of the World Medical Association (Declaration of Helsinki). Written consent was obtained from all participants after the study was explained to them. The protocol and informed consent documents were approved by the Institutional Review Board at Albert Einstein College of Medicine, Bronx, NY where the study was conducted.

### Stimuli

**Figure 1:**
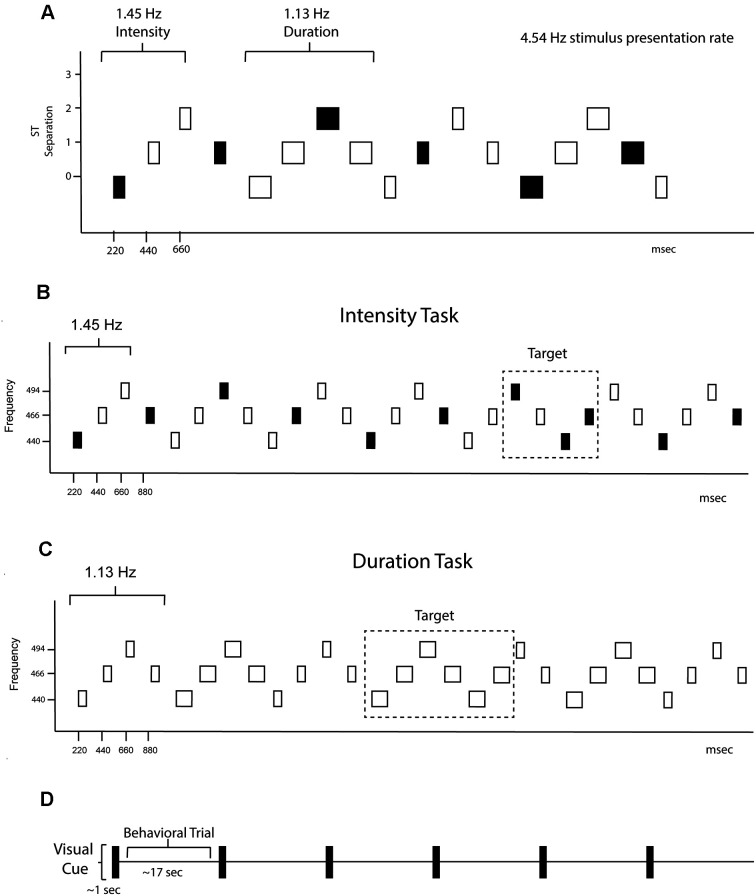
Experimental protocol. **(A)** Schematic of the overall stimulus paradigm. The X-axis shows the timing in milliseconds and the Y-axis shows the frequency separation in semitones (ST). The rectangles represent the tones. The fill represents intensity, with black representing louder intensity tones and the white softer intensity tones, and the width of the rectangle represents tone duration. Both intensity and duration patterns were presented randomly within the stimulus blocks and are demarcated with the rhythm of each denoted. The global rhythm of the tones in the sequence was 4.54 Hz. **(B)** Intensity task. Only the intensity rhythm is displayed. The standard 4-tone intensity pattern was loud-soft-soft-loud, and had a rhythm of 1.51 Hz. The task was to keep the standard pattern in mind and press the response key when the pattern deviant (loud-soft-**loud**-loud) was detected. Thus, the button press was time-locked to the 3rd tone of the pattern where a louder tone came when a softer tone was expected. The frequency of the tones is displayed on the y-axis. The tone duration changes were irrelevant to the intensity task. **(C)** Duration Task. Only the duration rhythm is displayed. The standard 8-tone duration pattern was short-short-short-short-long-long-long-long, and had a rhythm of 1.13 Hz. The task was to keep the standard pattern in mind and press the response key when the pattern deviant (long-long-long-long-**long**-long) was detected. Thus, the button press was time-locked to the 5th long tone of the deviant pattern where a shorter tone was expected. The frequency of the tones is displayed on the y-axis. The tone intensity changes were irrelevant during the duration task. **(D)** Protocol schematic. A visual cue the words “intensity task” or “duration task” (depicted in time as a black rectangle) was presented on the screen for 1 s instructing participants what task to do. Sounds were presented randomly between 15–20 s, comprising one trial. Six trials of “intensity task” or “duration task” were presented randomly throughout each of the 20 blocks.

A graphical representation of the sound sequence is shown in Figure 1. Stimuli consisted of pure tones presented binaurally through insert earphones with a constant stimulus onset asynchrony (SOA) of 220 ms. Every sequence consisted of a repeating four-tone frequency pattern in a set order 440 Hz—466.16 Hz—493.88 Hz—466.16 Hz (Figure 1A). There was one semitone separation between each successive tone, facilitating perceptual integration (Bregman, 1990). Eight tones created the duration pattern (Figure 1B), with four longer duration tones (200 ms each) followed by four shorter tones (100 ms each). Three tones created the intensity pattern (Figure 1C), one loud tone (85 dBA) followed by two soft tones (70 dBA; calibrated with a Brüel and Kjær^®^ sound level meter with an artificial ear). The resulting sequence from superimposing these patterns was perceptually ambiguous, with listeners able to hear either the rhythm of the intensity pattern (1.45 Hz) or the rhythm of the duration pattern (1.13 Hz). There were 12 total individual tones used in the sequences that accounted for all possible combinations of frequency (low/middle/high), duration (long/short), and intensity (loud/soft).

Randomly occurring violations in both the intensity and duration patterns (deviants) occurred for 15% of intensity pattern triplets, the standard *loud-soft-soft* pattern was replaced with *loud-soft-****loud*** (Figure 1B); and 15% of duration patterns, the standard *long-long-long-long-short-short-short-short* pattern was replaced with *long-long-long-long-****long-long****-short-short* (Figure 1C). The infrequent deviants served as targets during one half of the experiment and irrelevant deviants in the other half.

### Procedures

The two conditions, *Attend Visual* condition and *Attend Auditory* were conducted on two separate days. Participants were alternately assigned to start with one of the conditions and completed the other condition when they returned to the laboratory approximately 2 weeks later. In the *Attend Visual* condition, participants passively listened to the sound sequences while watching a closed-captioned movie (chosen from our video library). Ten 6-min sound sequences were presented, with each sequence having 1,458 stimuli (72 intensity deviants, 54 duration deviants).

In the *Attend Auditory* condition, participants listened to the sounds to identify the intensity and duration patterns and their deviants. The same set of sound sequences were used in both the *Attend Visual* condition and the *Attend Auditory* conditions but in a differently randomized order (20 sound sequences in all). A brief practice session was provided prior to the recording session. Participants were instructed about what sound patterns to listen for and were shown a visual depiction of the target patterns. Participants were then presented with a graded series of sound sequences to acquaint them with the task. First, they were presented with either the intensity or the duration pattern by itself (practicing one feature at a time with the order alternated across participants) and were instructed to identify the repeating pattern and press a response key when they heard violations to the pattern. Second, they were presented with the intensity or duration pattern along with the frequency modulation and asked to do the same task. Then, finally, they had the intensity, frequency, and duration modulations present and were told to focus on their target feature pattern (intensity or duration) and perform the same task they had been doing and ignore any other tone variations. After successfully training for one pattern feature task, they trained for the alternative task. 60% correct was used as the criterion used for both the intensity and the duration tasks to proceed to the EEG recording session. Two participants were excluded prior to data collection based on this practice criterion.

During the EEG recording, 20 differently randomized sequences were presented, each sequence having six trials of 15–20 s in length. Every trial was preceded by a visual stimulus that stayed on the monitor for 1 s to indicate which task intensity or duration patterns were to be performed on the next trial (Figure 1D). Only one of the tasks was performed for the duration of each trial. The total silent time between trials, including the visual stimulus, was 1.85 s. The time constant for decay of streaming bias is 1.42 s (Beauvois and Meddis, 1997). Thus, streaming bias persisted between trials. The tasks were split 50–50% among the trials so that on half of the trials participants performed the intensity task and half of the trials the duration pattern task. Task switching was also randomized so that half of the trials were “switching” trials (going from intensity task to duration task and vice versa) and half were “repeat” trials (repeating the same task as the previous trial). These two contingencies were orthogonal; thus one-quarter of trials were duration task switched (*Duration Switch*), one quarter were duration task repeated (*Duration Repeat*), one quarter were intensity task switched (*Intensity Switch*), and one quarter were intensity task repeated (*Intensity Repeat*). Regardless of which instruction was given, intensity and duration deviants were present in all trials, so participants needed to isolate and attend to one feature pattern (the *targets*) and ignore the distracting feature pattern (*non-targets*).

Participants sat in a comfortable chair in a sound-attenuated booth. The duration of one session, of which there were two occurring on separate days, was approximately 2 h, which included consenting, hearing screen, cap placement, task practice, task performance, and breaks.

### Data Analysis

#### Behavioral Responses

Target responses were calculated for Intensity and Duration deviants separately within a “switch” trial and a “repeat” trial for the four trial types: *Duration Switch, Duration Repeat, Intensity Switch, and Intensity Repeat*. A button press was considered correct when it fell within 100–900 ms from target onset. Reaction time (RT) was calculated as the mean RT of the correct responses. Hit rate (HR) was calculated as the number of correct button presses divided by the total number of target stimuli. A false alarm was considered a button press to a non-target deviant. The false alarm rate (FAR) was calculated as the number of button presses made within the response window for non-target deviants divided by the total number of non-target deviants. There was no overlap in the response windows. HR, RT, and FAR were reported separately for each trial type.

To evaluate task-switching effects, which are generally observed very soon after the switch, we separately analyzed the HR and RT to the first target in each of the trials from the remaining targets.

### EEG Recording and Data Reduction

Continuous EEG was recorded using a 32-channel electrode cap with the 10–20 international electrode placement system. An electrode placed at the base of the nose was used as the reference and the P09 electrode was used as the ground. Impedances were below 5 kΩ. EEG and EOG were sampled at a rate of 500 Hz using a bandpass filter of 0.05–100 Hz and a gain of 1,000 (Neuroscan Synamps amplifier, Compumedics Corp, El Paso, Texas). ERPs were extracted from the continuous EEG files. After applying a 0.1–30 Hz bandpass filter (using a finite impulse response filter with zero phase shift and a roll-off slope of 24 dB/octave), EEG data for each subject were separated into 700 ms epochs, including a 100-ms pre-stimulus interval. Ocular artifact correction was performed for an individual when excessive blinking resulted in the exclusion of more than 20% of trials. For three participants who had excessive eye-blink activity, ocular artifact reduction was conducted to perform the correction using Neuroscan EDIT software. This Singular Value Decomposition transform method is used to identify the blink component. From the continuous EEG, a file is created that reflects the spatial distribution of the blink and then used to remove the blink. The blink-corrected data were then baseline-corrected across the whole epoch (the mean was subtracted at each point across the epoch). After baseline correction, artifact rejection criteria were set to ±75 mV. On average, 87% of all trials were included in the analysis. Condition-matched deviant epochs and standard epochs were grouped accordingly then baseline corrected to the pre-stimulus period and averaged to create individual mean waveforms. Deviant epochs that contained a correct button press were marked as “Correct” and averaged together, incorrect responses were omitted from this averaged waveform. Individual mean averages were then averaged to create grand-mean waveforms. The grand-mean standard waveform was subtracted from the grand-mean deviant waveform from the same condition, yielding a grand-mean difference waveform used to identify ERP components. The mean latency of the MMN component in each condition was determined using the Neuroscan program to find the maxima between 100–300 ms at the left mastoid (LM) electrode in the grand mean difference waveform. LM was used to avoid overlap of attention components. The unattended deviants, both duration and intensity, showed a clear double peak, and both of these peaks were quantified. For the P3b component, the maxima were determined between 300–600 ms at the Pz electrode. The peak latency of each ERP component in the grand mean waveform was used as the center to obtain a 50 ms interval used to assess the amplitude of the MMN component and a 60 ms window to obtain the mean for each individual for the P3b component. MMN area was quantified as the area between the Fz electrode and the averaged mastoid electrodes [(LM + RM)/2] and P3b was quantified as the area under the Pz electrode.

To perform the frequency analysis to visualize the entrainment to the rhythm, regions around targets were removed from the continuous file (100 ms pre and 1,000 ms after) to remove contributions from target response and motor activity. A high pass filter at 0.5 Hz was used. Eyeblink correction was performed using Neuroscan LDR. Matching trials were concatenated and fast FFT was performed on the resultant continuous file. Frequency power was measured at the target rhythms (1.13 Hz for duration pattern and 1.45 Hz for intensity pattern) and the stimulus presentation rate (4.45 Hz and 9.10 Hz for the 1st harmonic). The normalized power was determined by dividing the power at a given frequency by the average power of the surrounding frequencies ±1 Hz, excluding the other target frequency (i.e., the average surround power for the 1.13 Hz frequency did not include 1.45 Hz and vice versa).

### Statistical Analyses

#### Behavioral Analyses

For HR and RT, separate repeated measures analysis of variance (rmANOVA) was performed with factors of task (Intensity/Duration), switching (switch/repeat), and primacy (first target/other targets). FAR was calculated using a two-way rmANOVA with factors of task and switching.

#### ERP Component Analyses

The first analysis determined the significant presence of the MMN and P3b components using a one-sided, one sample *t*-test to confirm that the amplitude was significantly greater than zero. The second analysis then compared the amplitude/latency of the ERP components across stimulus types and conditions. *Attend Auditory* condition: A four-way rmANOVA was used to compare the amplitude of the MMN with factors deviant type (Intensity vs. Duration), peak (First peak vs. Second peak), attention (attended vs. unattended), and switching (task switch vs. task repeat). “Attended” refers to MMNs elicited by the target stimuli and “unattended” refers to MMNs elicited by the non-target deviants. A second analysis was used to compare the unattended mean amplitude of the MMNs elicited by non-tartgets in the *Attend Auditory* condition to the mean amplitude MMNs elicited by deviants in the *Attend Visual* condition. A rmANOVA with factors of deviant type (Intensity/Duration), peak (First peak/Second peak), and trial type (Switch, Repeat, Attend Visual) was calculated.

P3b amplitude was compared using factors of task type (Intensity/Duration,) switching (task switch/task repeat), and electrode (Fz/Cz/Pz). Normal neural entrainment was analyzed with rmANOVA with factors of task type (Intensity/Duration) switching (task switch/task repeat), and rhythm (duration rhythm/intensity rhythm/stimulus rhythm/harmonic rhythm). A second analysis for neural entrainment was performed to compare the *Attend Auditory* conditions to the *Attend Visual* condition using rmANOVA with factors of conditions (Duration Switch/Duration Repeat/Intensity Switch/Intensity Repeat/Attend Visual) and rhythm(duration rhythm/intensity rhythm/stimulus rhythm/harmonic rhythm).

For all ANOVA calculations, where data violated the assumption of sphericity, degrees of freedom were corrected using Greenhouse-Geisser estimates of sphericity. Corrected *p* values are reported. For *post hoc* analyses, Tukey HSD for repeated measures was conducted on pairwise contrasts only when there were significant main effects or interactions. All statistical analyses were performed using Statistica software (Statsoft, Inc., Tulsa, OK, USA).

## Results

### Behavior

**Figure 2:**
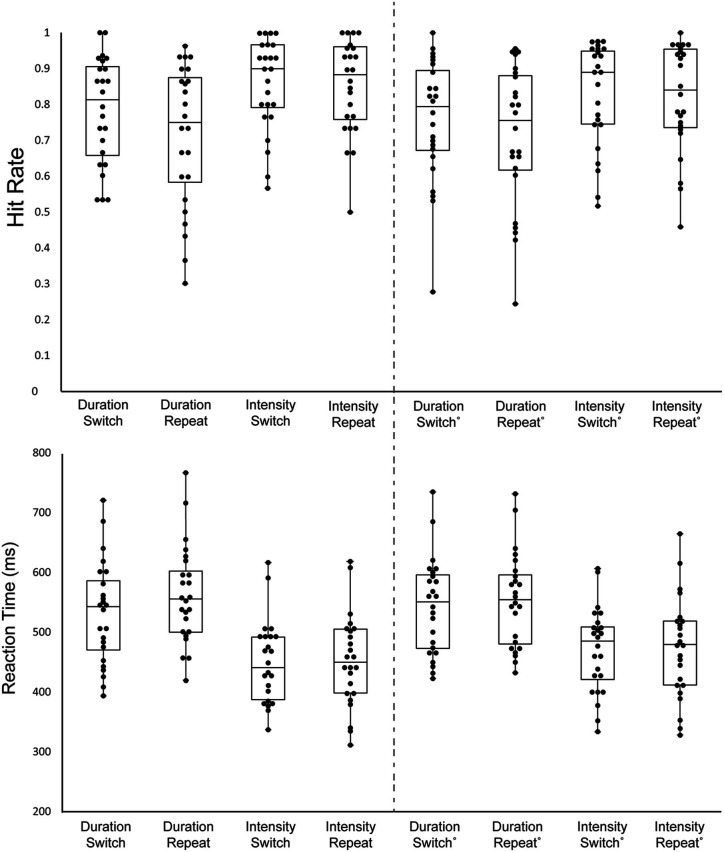
Behavioral results. Hit Rate (HR; top graph) and Reaction Time (RT; bottom graph) are displayed for all stimulus types. Each black circle represents one individual. The upper whisker denotes the upper limit, the lower whisker denotes the lower limit, the upper bar represents the 3rd quartile, the middle bar represents the median, and the lower bar represents the 1st quartile. Each column shows a different stimulus type, indicating whether it was the Duration or the Intensity task and whether the trial was a Switch trial (the previous trial was the other task) or a Repeat trial (the same task repeated). The left columns 1–4 display the mean data of the first target of the trials and the right columns 5–8 display the mean of the remaining targets in the trials.

Table 1 and Figure 2 display the behavioral results.

**Table 1:** Behavioral data.

Task	Hit rate		Reaction time (ms)		FAR
	First target	Other targets	First target	Other targets	All targets
**Duration/Switch**	0.78 (0.15)	0.76 (0.17)	532 (86)	543 (81)	0.020 (0.040)
**Duration/Repeat**	0.71 (0.20)	0.71 (0.20)	563 (82)	554 (79)	0.016 (0.023)
**Intensity/Switch**	0.86 (0.13)	0.83 (0.14)	449 (69)	470 (71)	0.027 (0.034)
**Intensity/Repeat**	0.85 (0.13)	0.82 (0.15)	453 (77)	473 (82)	0.021 (0.030)

*Standard deviation is shown in parenthesis. *ms*, milliseconds; *FAR*, false alarm rate*.

#### Hit Rate

There was a main effect of the order on HR, with the first target of a block having a higher HR than the average of the rest of the targets (*F*_(1,23)_ = 7.23, *p* = 0.01, $\eta_{p}^{2}$ = 0.24). There was a main effect of task on HR, with a higher HR for the Intensity Task (*F*_(1,23)_ = 8.71, *p* < 0.01, $\eta_{p}^{2}$ = 0.27). Additionally, there was a main effect of switching, with switch trials having higher HR than repeat trials (*F*_(1,23)_ = 7.23, *p* = 0.01, $\eta_{p}^{2}$ = 0.24). There was no interaction between order and task (*F*_(1,23)_ = 0.66, *p* = 0.42), between order and switching (*F*_(1,23)_ = 0.89, *p* = 0.36), between task and switching (*F*_(1,23)_ = 3.29, *p* = 0.08), or between order, task, and switching (*F*_(1,23)_ = 1.84, *p* = 0.19).

#### Reaction Time

There was a main effect of order on RT (*F*_(1,23)_ = 13.13, *p* < 0.01, $\eta_{p}^{2}$ = 0.36) with the first target of the block having a shorter RT. There was also a main effect of task (*F*_(1,23)_ = 45.58, *p* < 0.01, $\eta_{p}^{2}$ = 0.65), due to the shorter RT to intensity pattern targets than Duration pattern targets. Additionally, there was a main effect of switching (*F*_(1,23)_ = 11.9, *p* < 0.01, $\eta_{p}^{2}$ = 0.34), with switch trials having a shorter RT than repeat trials. There was an interaction between order and task (*F*_(1,23)_ = 14.59, *p* < 0.01, $\eta_{p}^{2}$ = 0.39). *Post hoc* calculation showed First Target-Duration>First Target-Intensity (*p* < 0.01), First Target-Duration> Later Targets-Intensity (*p* < 0.01) Later Targets-Duration>First Target-Intensity (*p* < 0.01), Later Targets-Intensity>First Target-Intensity (*p* < 0.01). This showed participants have a faster response to the first target for the intensity task when compared to later targets, but this does not hold true for the duration task. There was an interaction between, order and switching (*F*_(1,23)_ = 5.03, *p* = 0.03, $\eta_{p}^{2}$ = 0.18). *Post hoc* calculation showed First Target-Repeat>First Target-Switch (*p* < 0.01), Later Targets-Switch>First Target-Switch (*p* < 0.01), Later Targets-Repeat>First Target-Switch (*p* < 0.01). Participants had the fastest response time to the first target after a task switch when compared to other target types. There was no interaction between task and switching (*F*_(1,23)_ = 3.55, *p* = 0.07). There was a three-way interaction between order, task, and switching (*F*_(1,23)_ = 5.23, *p* = 0.03, $\eta_{p}^{2}$ = 0.19). *Post hoc* analysis showed First Target-Duration-Repeat>First Target-Duration-Switch (*p* < 0.01), First Target-Duration-Switch>First Target-Intensity-Switch (*p* < 0.01), First Target-Duration-Switch>First Target-Intensity-Repeat (*p* < 0.01), Later Targets-Duration-Repeat>First Target-Duration-Switch (*p* < 0.01), First Target-Duration-Switch>Later Targets-Duration-Repeat (*p* < 0.01).

#### False Alarm Rate

False alam rate (FAR) did not differ as a function of the task being performed or whether it was a switch or repeat trial. There was no main effect of task (*F*_(1,23)_ = 1.44, *p* = 0.24), no main effect of switching (*F*_(1,23)_ = 3.73, *p* = 0.07), and no interaction between task and switching (*F*_(1,23)_ < 1, *p* = 0.90).

### Event Related Brain Potentials

#### MMN

**Figure 3:**
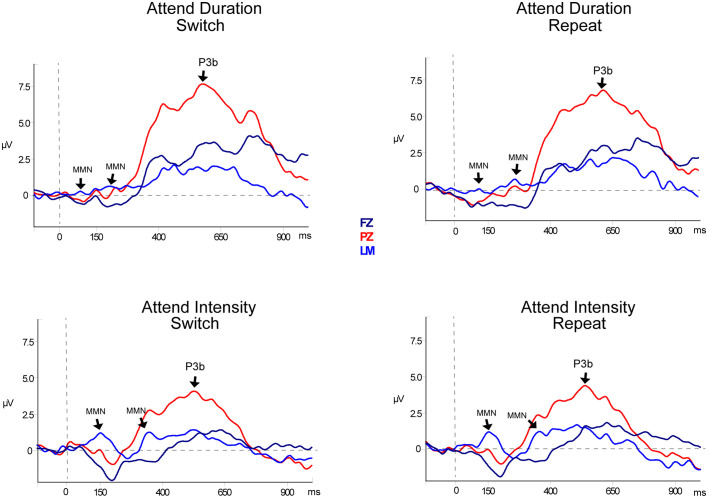
Event-related potentials for target pattern deviants. Difference waveforms (deviant-minus-standard) are displayed for duration (top row) and intensity (bottom row) targets. Responses to the target during Switch trials are shown in the left column and responses to the targets during Repeat trials are shown in the right column. Responses recorded from Fz (dark blue solid line), the left mastoid (LM; light blue solid line), and Pz (red solid line) are overlain to demarcate both the mismatch negativity component (MMN) response and the P3b components. Significant components are denoted with an arrow and labeled. Two successive MMNs were elicited by two successive tones within the pattern deviants.

**Figure 4:**
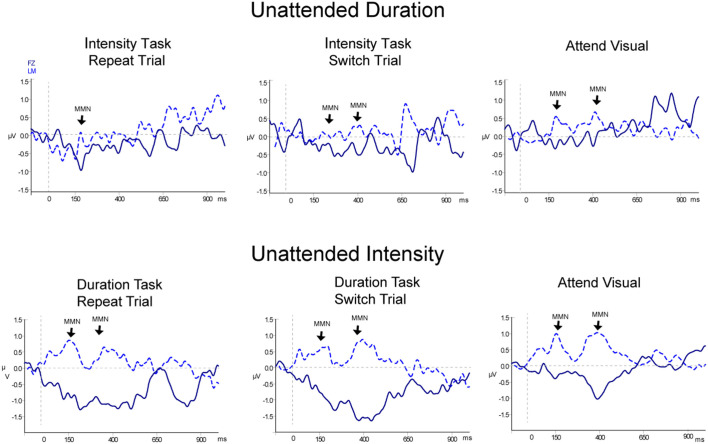
Event-related potentials for non-target (unattended) pattern deviants. Difference waveforms (deviant-minus-standard) are displayed for duration (top row) and intensity (bottom row) non-targets. Responses to the non-targets during the Attend Auditory conditions: Repeat trials are shown in the left column and Switch trials are shown in the middle column, and responses to the non-targets in the Attend-Visual condition are displayed in the right column. The dark blue solid line displays the waveform recorded from the Fz electrode with the waveform at LM (light blue dashed line) overlain. Significant MMN components are denoted with an arrow pointed at LM and labeled. Two successive MMNs were elicited by two successive tones within the non-target pattern deviants, similarly as for the targets shown in Figure 3.

Table 2, Figures 3 and 4 display the MMN results. MMNs were elicited by the first two tones of the intensity and duration deviant patterns for both attended (Figure 3 labeled with arrows) and unattended (Figure 4, labeled with arrows) pattern deviants (determined by one-sample *t*-tests all *p* < 0.05). For example, when *long-long-long-long-short-short-short-short* pattern was replaced with *long-long-long-long-****long-long****-short-short*, detection of the deviant could occur at the 5th long tone but both of the longer duration tones were deviant within the 8-tone pattern. The second deviant tone of the unattended duration pattern when the intensity task was being performed was the only second deviant that did not elicit MMN (*p* = 0.13).

**Table 2:** ERP amplitudes.

Condition	Task	MMN component				P3b component	
		Dur 1	Dur 2	Int 1	Int 2	Duration	Intensity
Attend/Auditory	Duration/Switch	−0.72 (1.28)	−1.11 (1.57)	1.35 (1.19)	−2.43 (1.61)	6.68 (3.70)	–
Attend/Auditory	Duration/Repeat	−0.90 (0.92)	−1.34 (0.19)	−1.63 (1.23)	−1.53 (1.08)	6.01 (3.55)	–
Attend/Auditory	Intensity/Switch	−0.49 (0.83)	−0.45 (0.84)	−2.35 (1.47)	−1.87 (2.36)	–	3.10 (2.71)
Attend/Auditory	Intensity/Repeat	−0.47 (0.81)	−0.33 (1.03)	−2.29 (1.13)	−1.48 (2.57)	–	3.77 (2.73)
Attend/Visual	Watching movie	−0.59 (0.52)	−0.66 (0.67)	−1.05 (1.06)	−1.96 (0.94)	–	–

*Standard deviation is shown in parentheses. *Dur1*, duration pattern deviant first peak; *Dur2*, duration pattern deviant second peak; *Int1*, intensity pattern deviant first peak; *Int2*, intensity pattern deviant second peak; – no ERP component elicited*.

In the Attend Auditory Condition, the MMN amplitude elicited by intensity pattern deviants was larger than duration pattern deviants (main effect of deviant type, *F*_(1,23)_ = 32.27, *p* < 0.01, $\eta_{p}^{2}$ = 0.79). MMN amplitude was larger for the attended compared to the unattended tone pattern features (main effect of attention, *F*_(1,23)_ = 5.19, *p* = 0.03, $\eta_{p}^{2}$ = 0.18). MMNs elicited by the first two tones did not differ in amplitude (no main effect of the peak, *F*_(1,23)_ = 0.05, *p* = 0.82, $\eta_{p}^{2}$ < 0.01), nor did it matter if it was a switch or repeat trial (no main effect of switching, *F*_(1,23)_ = 0.64, *p* = 0.43, $\eta_{p}^{2}$ = 0.02). There was an interaction between peak and switching (*F*_(1,23)_ = 7.27, *p* = 0.01, $\eta_{p}^{2}$ = 0.24). *Post hoc* calculations showed that this was due to the MMN to the second tone of the pattern being larger in the switch trials than the second peak of the repeat trials (*p* = 0.05). There was a significant three-way interaction between deviant type, peak, and attention (*F*_(1,23)_ = 13.72, *p* < 0.01, $\eta_{p}^{2}$ = 0.37). *Post hoc* calculations showed that Duration-First Peak-Attended < Intensity-First Peak-Attended (*p* < 0.01), Duration-First Peak-Attended < Intensity-Second Peak-Attended (*p* = 0.01), Duration-First Peak-Attended < Intensity-Second Peak-Unattended (*p* < 0.01). There was a three-way interaction between deviant type, peak, and switching (*F*_(1,23)_ = 8.30, *p* < 0.01, $\eta_{p}^{2}$ = 0.27). *Post hoc* analysis showed that the intensity MMN elicited by the second tone of the deviant pattern when it was a switch task was smaller in amplitude than the other intensity MMN peaks. There were no interactions between deviant type and peak (*F*_(1,23)_ < 1, *p* = 0.44), between deviant type and attention (*F*_(1,23)_ = 0.99, *p* = 0.33), between peak and attention (*F*_(1,23)_ = 2.48, *p* = 0.13) between deviant type and switching (*F*_(1,23)_ = 2.07, *p* = 0.16), between attention and switching (*F*_(1,23)_ = 0.68, *p* = 0.42), between deviant type, attention, and switching (*F*_(1,23)_ = 0.27, *p* = 0.61), between peak, attention, and switching (*F*_(1,23)_ = 3.68, *p* = 0.07), or between deviant type, peak, attention, and switching (*F*_(1,23)_ = 1.29, *p* = 0.27).

When all of the sounds were unattended, in the *Attend Visual* condition, as compared to when they were unattended in the *Attend Auditory* condition, intensity deviants elicited a larger amplitude MMN than duration deviants (the main effect of deviant type, *F*_(1,23)_ = 47.82, *p* < 0.01, $\eta_{p}^{2}$ = 0.68). The MMN elicited by the second tone of the pattern was larger in amplitude than the first (main effect of the peak, *F*_(1,23)_ = 6.79, *p* = 0.02, $\eta_{p}^{2}$ = 0.23). Unattended MMNs did not differ in amplitude as a function of whether auditory or visual was attended. There was no main effect of condition (*F*_(1,46)_ = 0.64, *p* = 0.53). There was a significant interaction between deviant type and peak (*F*_(1,23)_ = 8.60, *p* < 0.01, $\eta_{p}^{2}$ = 0.27). *Post hoc* showed Duration-First Peak < Intensity-First Peak (*p* < 0.01), Duration-First Peak < Intensity-Second Peak (*p* < 0.01), Duration-Second Peak < Intensity-First Peak (*p* < 0.01), Duration-Second Peak < Intensity-Second Peak (*p* < 0.01), and Intensity-First Peak < Intensity-Second Peak (*p* < 0.01). There was also an interaction between peak and condition (*F*_(1,46)_ = 8.30, *p* < 0.01, $\eta_{p}^{2}$ = 0.26). *Post hoc* calculation revealed that the 2nd MMN peak in the Attend Intensity and Attend Visual conditions were larger in amplitude than the MMNs in Attend Duration. There was no interaction between deviant type and condition (*F*_(1,46)_ = 1.97, *p* = 0.15). There was a three-way interaction between deviant type, peak, and condition (*F*_(1,46)_ = 13.72, *p* < 0.01, $\eta_{p}^{2}$ = 0.37). *Post hoc* calculations showed that the intensity MMN at the first peak was smaller in the Attend Visual condition than all of the other MMN peaks.

#### P3b Component

P3b amplitude was largest when performing the duration task (main effect of task, *F*_(1,23)_ = 24.67, *p* < 0.01, $\eta_{p}^{2}$ = 0.52). There was a significant main effect of electrode (*F*_(1,46)_ = 65.77, *p* < 0.01, $\eta_{p}^{2}$ = 0.74). *Post hoc* calculation showed Fz < Cz (*p* < 0.01) and Fz < Pz (*p* < 0.01). Amplitude did not differ between switching and repeat trials (no main effect of switching, *F*_(1,23)_ = 0.18, *p* = 0.07,). There was a significant interaction between deviant type and switching (*F*_(1,23)_ = 6.15, *p* = 0.02, $\eta_{p}^{2}$ = 0.21). *Post hoc* calculations showed that Duration Repeat<Duration Switch (*p* < 0.01), Intensity Repeat < Duration Repeat (*p* < 0.01), Intensity Switch < Duration Repeat (*p* < 0.01), Intensity Repeat < Duration Switch (*p* < 0.01), and Intensity Switch < Duration Switch (*p* < 0.01). There was a significant interaction between deviant type and electrode (*F*_(1,46)_ = 15.5, *p* < 0.01, $\eta_{p}^{2}$ = 0.40). *Post hoc* calculations show that the duration deviant P3b amplitude was larger than intensity deviant P3b at Cz and Pz electrodes, with no difference in amplitude between them at Fz. There was no interactions between switching and electrode (*F*_(1,46)_ = 1.16, *p* = 0.32, $\eta_{p}^{2}$ = 0.05), and or between deviant type, switching, and electrode (*F*_(1,23)_ < 1, *p* = 0.67).

#### Rhythmic Entrainment

**Figure 5:**
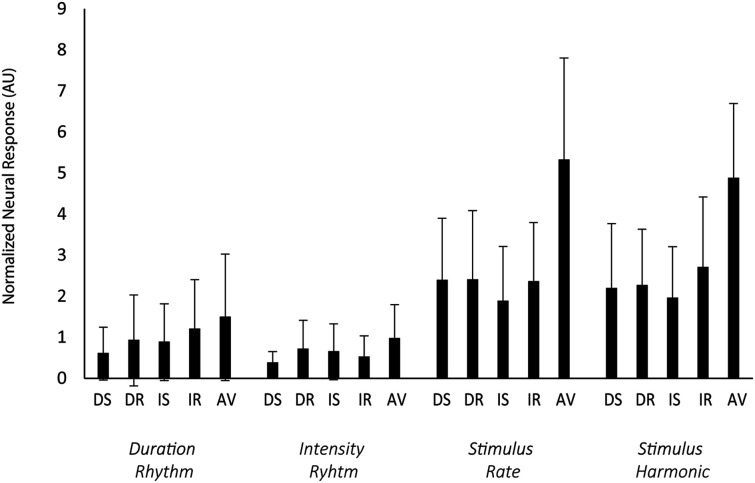
Rhythmic entrainment. Rhythmic entrainment responses to the standard patterns are shown as the normalized neural response from the Cz electrode (y-axis), calculated as the square of the power of target frequency divided by the square of the average power in the surrounding 1 Hz frequency bin (excluding other target frequencies). Error bars display the standard deviation. Along the x-axis, the blue rectangles represent each task trial type (DS, duration switch trial; DR, duration repeat trial; IS, intensity switch trial; IR, intensity repeat trial; AV, attend visual stimuli). The sub x-axis is separated by duration rhythm (1.13 Hz), intensity rhythm (1.45), overall stimulus rate (4.45 Hz), and the first harmonic of the stimulus rate (9.10 Hz). The direction of attention had no effect on the strength of the responses. Attended rhythms in the Attend Auditory condition were not distinguished from unattended rhythms in the Attend Auditory condition.

Figure 5 shows the normalized neural responses to rhythmic entrainment. The raw data are presented in Supplementary Figure 1 (Supplementary Material). Neural responses did not differ as a function of the task performed (no main effect of task (*F*_(1,23)_ = 0.10, *p* = 0.75). Repeat trials had greater normalized power than switching trials (main effect of switching *F*_(1,23)_ = 5.97, *p* = 0.02, $\eta_{p}^{2}$ = 0.21). There was also a main effect of rhythm, with the global stimulus rate and first harmonic (4.55 Hz and 9.10 Hz) both having greater power than the power of an individual feature pattern (1.14 Hz or 1.45 Hz; *F*_(3,69)_ = 45.55, *p* < 0.01, $\eta_{p}^{2}$ = 0.66). There was no interaction between task and switching (*F*_(1,23)_ < 1, *p* = 0.41), no interaction between task and rhythm (*F*_(3,69)_ < 1, *p* = 0.41), no interaction between switching and rhythm(*F*_(3,69)_ < 1, *p* = 0.74), and no three-way interaction between task, switching, and rhythm (*F*_(3,69)_ = 1.08, *p* = 0.036).

There was a significant effect of condition (*F*_(4,92)_ = 35.54, *p* < 0.01, $\eta_{p}^{2}$ = 0.61). Power was greater in the *Attend Auditory* than *Attend Visual* condition. There was also a main effect of rhythm (*F*_(3,69)_ = 61.45, *p* < 0.01, $\eta_{p}^{2}$ = 0.73) with greater power to the global rhythm than either of the individual feature rhythms and interaction between condition and rhythm (*F*_(12,276)_ = 6.67, *p* < 0.01, $\eta_{p}^{2}$ = 0.22). The power in the global rhythms (4.55 Hz and 9.10 Hz) was greater when the auditory stimuli were attended, greater in the *Attend Auditory* than *Attend Visual*.

## Discussion

The current study varied rhythmic attention to two non-overlapping tone feature patterns to investigate how memory would represent sound patterns when multiple rhythmic interpretations could be perceived from a single sound stream. Although we expected that attending to one rhythm to perform a task would dampen the brain’s response to the alternate rhythm, we found this not to be true. Evidence from neural entrainment to the target rhythm (the standard pattern) and by MMN elicited to pattern violations (deviance detection) demonstrated that both intensity and duration rhythms were maintained in memory irrespective of the direction of attention. The normalized power to the attended rhythm was not differentiated from the unattended rhythm and MMNs were elicited by pattern violations in the attended and unattended rhythms. Thus, attention to one pattern did not modulate the representation of the unattended, distracting pattern. Both patterns were tracked simultaneously in memory despite task goals.

### Attention Effects and Multitasking

The presence of MMN, elicited by both of the pattern deviants, indicates that both feature patterns were distinctly represented in working memory. There were no amplitude differences in the MMN elicited in the *Attend Auditory* condition by attended targets and unattended auditory pattern deviants, and the *Attend Visual* condition to unattended deviants for both feature patterns. The MMN amplitude was larger for the intensity pattern deviants, suggesting the intensity task was either easier or more physically discriminable than the duration pattern deviants (Näätänen and Alho, 1995). Based on research with bistable visual stimuli, one might expect that maintaining one feature pattern to perform a task could suppress representation of the other feature pattern, to minimize task interference. However, that did not appear to be the case. Performing one task did not result in suppression of the alternative percept as MMNs were elicited by both attended feature pattern targets and by unattended feature pattern deviants. These results differ from our previous study (Costa-Faidella et al., 2017) that used a similar paradigm and found enhanced power to the attended rhythm compared to the unattended rhythm. However, there were some distinct differences that may explain the difference in our results. First, the Costa-Faidella et al. (2017) study did not use a switching paradigm, participants performed the same task in blocks of 12 trials before switching to another task. Second, the task did not involve pattern detection. Participants counted the number of stimuli occurring in the block. Finally, there were no pattern violations in the attended or unattended patterns. Selective entrainment was calculated based on tracking the attended pattern. Thus, one explanation for finding representation of both feature patterns (without enhancement of the attended) is that this was a switching task with participants alternating randomly 50–50% between doing the intensity pattern and duration pattern tasks within each stimulus block. As such, both percepts had to be “active” to efficiently perform the tasks when the unexpected visual cue instructed which pattern to attend to and detect deviants. Another explanation is that the pattern deviants themselves may have evoked some attentional (passive) awareness to the unattended pattern, which may have negated any enhancement to the attended. That is, the brain was multitasking between attended and unattended pattern deviance detection (Miller et al., 2015; Sussman, 2017; Symonds et al., 2020; Brace and Sussman, 2021).

One of the goals was to evaluate how attention modified sound representations of the standards and deviants. Thus, in addition to analyzing the brain response to the unattended standard and unattended deviant patterns when attention was directed to one of the two, we also recorded the brain response when attention was directed to watching a movie and neither pattern was attended, the sounds were irrelevant to the task. We expected to find a difference between the unattended feature patterns when comparing the *Attend Auditory* and *Attend Visual* conditions. However, this was not the case. Having no task with the sounds did not dampen the response to the unattended sounds compared to when one sound feature was attended, and one sound feature unattended. This indicates that attention to the sounds is not necessary for the two feature patterns to be simultaneously tracked and represented in working memory. MMNs of similar amplitudes and latencies were elicited by both of the unattended feature pattern deviants and entrainment to the individual rhythms was maintained in the *Attend Visual* condition. However, some further exploration may clarify the difference between having fluctuation in frequency, duration, and intensity occur simultaneously or sequentially. In this experiment, we varied frequency, duration, and intensity parameters in sequential patterns of sounds. In realistic scenarios sounds sometimes vary simultaneously along multiple dimensions such as envelope, location, and other spectral components. Future studies may address how the auditory system tracks sequential vs. simultaneous variations of the auditory features.

### Neural Entrainment

We were initially surprised to find no task-dependent enhancement of the target rhythm frequencies based on previous studies (Mesgarani and Chang, 2012; Costa-Faidella et al., 2017). The relative power to the individual feature rhythms did not differ for any of the attentional manipulations and there was no enhancement or suppression based on switch and repeat trials. It was clear through the rhythmic entrainment to the standard patterns, however, that there was maintenance of both simultaneously. This maintenance of the standard for both feature patterns is consistent with the MMN results of the study, with MMN amplitudes elicited by pattern deviants similarly to both patterns regardless of task demands. From this perspective, it is not surprising that both standard rhythms were similarly represented with equal power, unrelated to task demands. That is, finding entrainment to the rhythm of both standard patterns is consistent with finding MMNs elicited by attended and unattended pattern deviants.

### Switch vs. Repeat Trials

HR was higher and RT shorter to the first target of the trial and higher after a task switch compared to the first target of the repeated trial. This is somewhat surprising on the surface based on task switching paradigms that commonly report a switching cost, lower HR, and longer RT, to the first trials after a task switch. However, our paradigm has not previously been used before and there are some differences that may distinguish the type of attention needed for preparing to perform one task or the other. A cue is provided to initiate the task that is then repeated through several trials before another cue is presented to either repeat or switch the task set. Thus, vigilance at the beginning of the trial may be greater when switching task is set than when repeating. This may explain why the initial trial of the repeat blocks showed a performance “cost.” The readiness may have been biased toward switching tasks whenever a visual cue was presented and maintaining the task set may have taken more adjustment time. Another possibility is that because it is a bistable stimulus sequence, one sequence can be perceived in two different ways depending on what you focus on, maintaining one of the two possible percepts may take more effort if there is a propensity for spontaneous switching during the presentation of a seconds-long sequence. In this view, switching tasks would be easier to do than maintaining the previous task because maintaining one perceptual organization involves overcoming the propensity to switch to the other percept. This might have resulted in a longer RT or more missed trials for repeat trials. Performing the same task repeatedly with bistable stimuli may be more difficult than switching between the two percepts when the stimuli are ambiguous (Denham et al., 2013).

Another explanation addresses the difference in processing between the cue and the task (Allport and Wylie, 1999; Grange and Houghton, 2010). For the current paradigm, the time interval from the visual cue to the first target may be long enough that there is no interference between processing the cue and the time it takes participants to switch task sets. The silent period between trials may also have facilitated the ease of switching tasks. Additionally, the P3b amplitude did not distinguish switch vs. repeat trials, which may be consistent with the distinction between these phases, with no task interference. The P3b amplitude differed between tasks, with a smaller P3b amplitude for the intensity task, consistent with the interpretation that the intensity task was easier than the duration task and thus required less effort to perform (Kok, 2001).

## Conclusion

The most critical finding of this experiment, shown by evidence from the standard and the deviant patterns, was that multiple, independent sound feature patterns (duration and intensity) were processed simultaneously despite the deployment of attention to task switching, task repeating, or watching a movie. The two neural indices that demonstrated this were: (1) neural entrainment to the standard patterns; and (2) the MMN components elicited by pattern deviants. Neural representations were similarly robust despite the direction of attention or task load. Normalized power to the rhythm of the standard attended feature patterns was similar to the rhythm of the standard unattended feature patterns. Additionally, the MMN was elicited by task-based feature pattern deviants with a similar amplitude as MMNs elicited by unattended feature patterns that were unattended because they were not the target pattern or because the participant ignored the sounds and watched a movie. Thus, the present data demonstrate a high level of adaptability and flexibility of the auditory system to navigate complex scenes when there are competing sound events. Results suggest a type of “multitasking” of the auditory system between attended and unattended sounds. That is, attending to one sound event does not negate representation of other sound events. This ability to track both attended and unattended regularities may be a crucial process involved in task-switching in complex sound environments.

## Data Availability Statement

The raw data supporting the conclusions of this article will be made available by the authors, without undue reservation.

## Ethics Statement

The study was carried out in accordance with the Code of Ethics of the World Medical Association (Declaration of Helsinki). Written consent was obtained from all participants after the study was explained to them. The protocol and informed consent documents were approved by the Institutional Review Board at Albert Einstein College of Medicine, Bronx, NY where the study was conducted.

## Author Contributions

KB: conceptualization, data curation, formal analysis, investigation, methodology, and writing—original draft. ES: conceptualization, formal analysis, funding acquisition, methodology, supervision, visualization, writing—review and editing. All authors contributed to the article and approved the submitted version.

## Conflict of Interest

The authors declare that the research was conducted in the absence of any commercial or financial relationships that could be construed as a potential conflict of interest.

## Publisher’s Note

All claims expressed in this article are solely those of the authors and do not necessarily represent those of their affiliated organizations, or those of the publisher, the editors and the reviewers. Any product that may be evaluated in this article, or claim that may be made by its manufacturer, is not guaranteed or endorsed by the publisher.
