# Supplementary figures and images for: The Brain Tracks Multiple Predictions About the Auditory Scene
Source: Front Hum Neurosci. 2021 Nov 3;15:747769. doi: 10.3389/fnhum.2021.747769 (PMC8595267; doi:10.3389/fnhum.2021.747769)

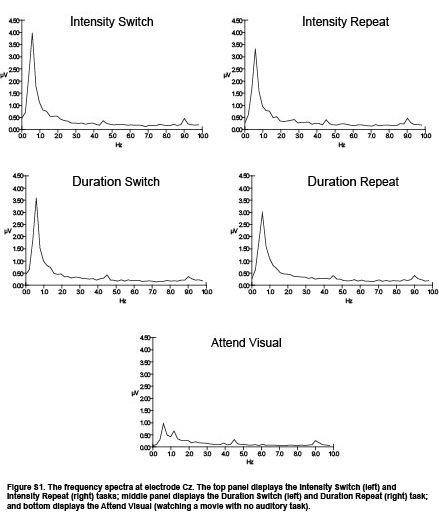

Supplement: Supplementary file 1 [file Image_1.JPEG]
